# Physiological and immunological barriers in the lung

**DOI:** 10.1007/s00281-024-01003-y

**Published:** 2024-03-07

**Authors:** Takahiro Kageyama, Takashi Ito, Shigeru Tanaka, Hiroshi Nakajima

**Affiliations:** 1 Department of Allergy and Clinical Immunology, Graduate School of Medicine, Chiba University, 1-8-1 Inohana, Chiba, 260-8670 Japan; 2 Institute for Advanced Academic Research, Chiba University, Chiba, Japan; 3 Chiba University Synergy Institute for Futuristic Mucosal Vaccine Research and Development (cSIMVa), Chiba, Japan

**Keywords:** Physiological barriers, Immunological barriers, Lung, Asthma

## Abstract

The lungs serve as the primary organ for respiration, facilitating the vital exchange of gases with the bloodstream. Given their perpetual exposure to external particulates and pathogens, they possess intricate protective barriers. Cellular adhesion in the lungs is robustly maintained through tight junctions, adherens junctions, and desmosomes. Furthermore, the pulmonary system features a mucociliary clearance mechanism that synthesizes mucus and transports it to the outside. This mucus is enriched with chemical barriers like antimicrobial proteins and immunoglobulin A (IgA). Additionally, a complex immunological network comprising epithelial cells, neural cells, and immune cells plays a pivotal role in pulmonary defense. A comprehensive understanding of these protective systems offers valuable insights into potential pathologies and their therapeutic interventions.

## Introduction

In humans, the respiratory tract is divided into the upper airways (nasal cavity, pharynx, and larynx) and the lower airways (trachea, bronchi, bronchioles, and alveoli). The lungs consist of the bronchi, bronchioles, and alveoli. The primary function of the respiratory tract is to efficiently change gas between inhaled air and the bloodstream [1]. The lungs are constantly exposed to various environmental elements, including pathogens, toxins, and allergens, that cause pulmonary infections or inflammation. Therefore, the human body needs to defend itself against countless intruders through the respiratory tract. Thus, the respiratory system has developed highly sophisticated barrier functions through physiological and immunological mechanisms. Airway epithelial cells (AECs) were originally thought to serve only as a physical barrier. However, recent research has elucidated the interplay between AECs and immune cells, revealing that AECs initiate immune responses [2]. This complex network of epithelial and immune cells is involved in the pathogenesis of various diseases [3–8]. In fact, inhibitors of thymic stromal lymphopoietin (TSLP), a cytokine mainly produced by AECs in the lungs, have emerged as therapeutic agents for asthma [9]. Furthermore, there is growing interest in the tripartite network of epithelium, immune, and neural cells [10–14]. In this review, we summarize how the lungs establish sophisticated barrier mechanisms.

### Multi-layered lung barriers

The barrier mechanism of the lung is, first, a continuous layer lining the respiratory tract composed of AECs, separating the body from the environment. Second, mucociliary clearance (MCC), consisting of the production of mucus and the coordinated movement of cilia, is the defense mechanism. Third, antimicrobial peptides and proteins act as a chemical barrier. Fourth, various cells, including both immune and non-immune cells, work in a coordinated manner to establish an immunological barrier. By summarizing the function of AECs and innate immune cells as barriers, we will attempt to understand the defense mechanisms in the lung and their involvement in pulmonary diseases.

## Physicochemical barriers

### Cell adhesion

**Fig. 1:**
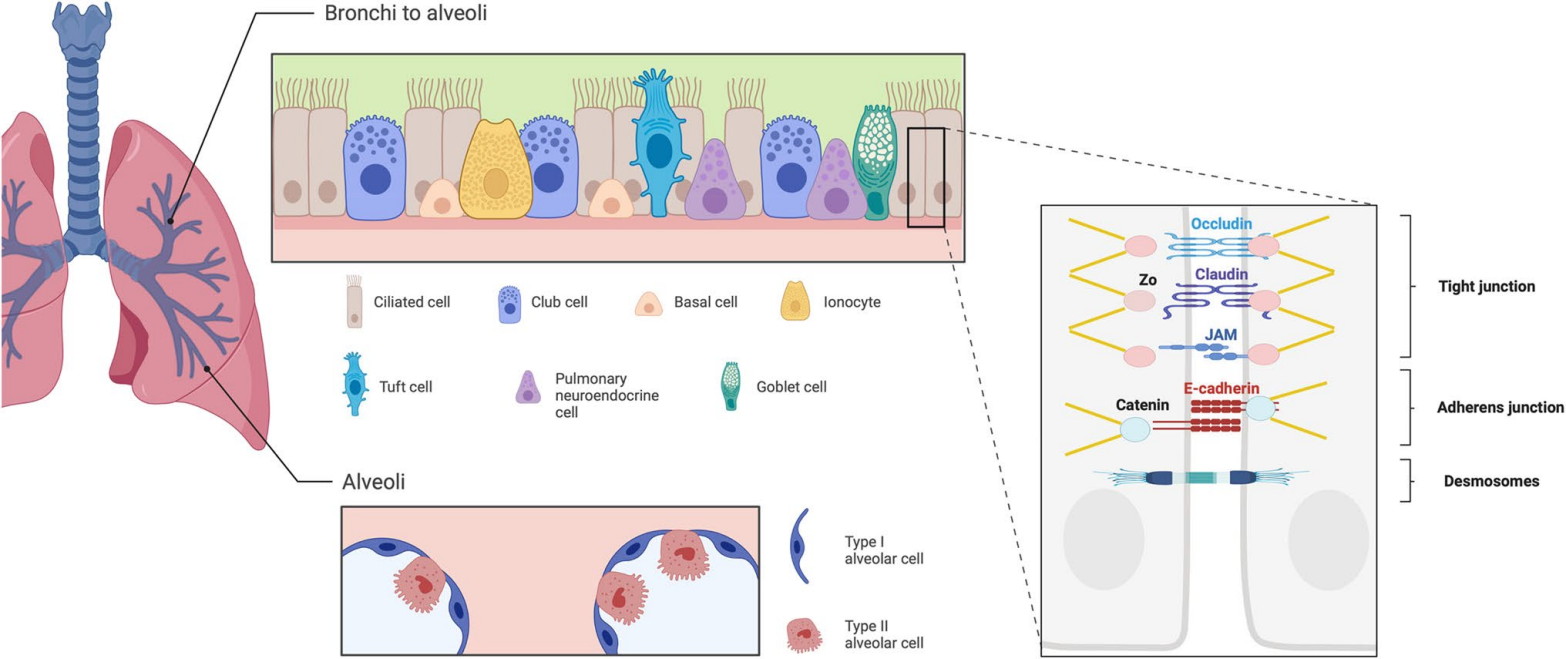
Bronchial and alveolar epithelial cell types and intercellular adhesion in the respiratory epithelium. The bronchial epithelium is composed of basal cells, ciliated cells, club cells, goblet cells, pulmonary endocrine cells (PNECs), tuft cells, and ionocytes. Basal cells can differentiate into other AECs as well as self-renew. Ciliated cells play a major role in mucociliary clearance (MCC) by moving mucus. Club cells secrete anti-inflammatory proteins such as Scgb1a1. Goblet cells are critical for mucus production. Ionocytes express high levels of CFTR, which is thought to play a role in maintaining the hydration and pH of the airway. Alveolar epithelium is composed of AT1 cells and AT2 cells. AT1 cells are essential for gas exchange and barrier function, while AT2 cells produce surfactant and GM-CSF. AT2 cells also function as progenitor cells. Cell–cell adhesion complexes mainly consist of tight junctions (TJs), adherens junctions (AJs), and desmosomes. TJs are composed of occludin, claudin, and junctional adhesion molecules (JAMs), which adhere cell to cell, and each binds to zonula occludens (ZO) proteins intracellularly and then connects to actin fiber. TJs primarily regulate paracellular permeability. AJs are cadherin-catenin complexes located below TJs, linking to the actin cytoskeleton. AJs control cell morphology and kinetics. Desmosomes bind intermediate filaments intracellularly to consolidate mechanical stability

The respiratory epithelium begins as a pseudostratified columnar epithelium in the nasal cavity, trachea, and bronchi, transitioning into columnar and cuboidal cells in the bronchioles. Finally, it forms a thin single-cell alveolar epithelium in the alveoli. The bronchial and alveolar epitheliums are composed of different cell types (Fig. 1). Bronchial epithelial cells consist of basal cells, ciliated cells, goblet cells, club cells, tuft cells, neuroendocrine cells, and ionocytes [15]. Alveolar cells can be divided into alveolar type 1 epithelial cells (AT1) and alveolar type 2 epithelial cells (AT2) [16].

The effectiveness of the physical barrier is a result of the coordinated interaction between neighboring epithelial cells through cell–cell adhesion complexes. The connection between adjacent airway epithelial cells is facilitated by apical tight junctions (TJs), adherens junctions (AJs), and desmosomes. They selectively regulate paracellular permeability, limit the transport of macromolecules, and maintain barrier integrity [17].

TJs are the uppermost intracellular junctions and play a pivotal role in regulating paracellular permeability. Using TJs, AECs can selectively control the passage of substances, particularly ions, water, and macromolecules. TJs comprise several essential transmembrane proteins of the claudin family, occludin, tricellulin, and junctional adhesion molecules (JAMs). The claudin family mainly regulates tight junction permeability. Additionally, there are critical cytoplasmic proteins associated with TJs, including zonula occludens (ZO)-1, ZO-2, and ZO-3, which bind directly to the transmembrane proteins, including claudins and occludin, on one end and connect to actin cytoskeleton on the other end [18, 19].

AJs are cadherin-catenin adhesion complexes located below TJs. AJs primarily provide mechanical strength by mediating adhesion between neighboring cells. In addition, AJs are involved in the establishment and maintenance of cell polarity in AECs, which is vital for proper tissue organization and function. The major component of AJs is transmembrane protein E-cadherin: its extracellular domain binds homotypically to adjacent cells, while the cytoplasmic domain binds to the catenins, linking to actin cytoskeleton. Through these networks, AJs regulate cell shape and movement and transmit mechanical forces between the cells [20].

Desmosomes are located around the midpoint of epithelial cells, providing robust mechanical stability. Desmosomes consist of transmembrane proteins, including desmogleins and desmocollins, that interact with intracellular proteins, such as desmoplakin, to anchor intermediate filaments. Hemidesmosomes, on the other hand, assist in anchoring the epithelial layer to the basal membrane, contributing to tissue integrity [17].

The robust cell adhesion described above is crucial not only as a barrier function but also for maintaining respiratory homeostasis. On the contrary, the disruption of this critical function is implicated in numerous pathological conditions. It has been observed that E-cadherin and ZO-1 expression is decreased, and allergen permeability is increased in AECs of asthmatic patients [21, 22]. Additionally, cigarette smoke may contribute to the pathogenesis of chronic obstructive pulmonary disease (COPD) by impairing cell adhesion in AECs [23, 24]. However, whether the dysfunction of cell adhesion is the etiology or the result of diseases must be interpreted with caution.

### Mucociliary clearance

**Fig. 2:**
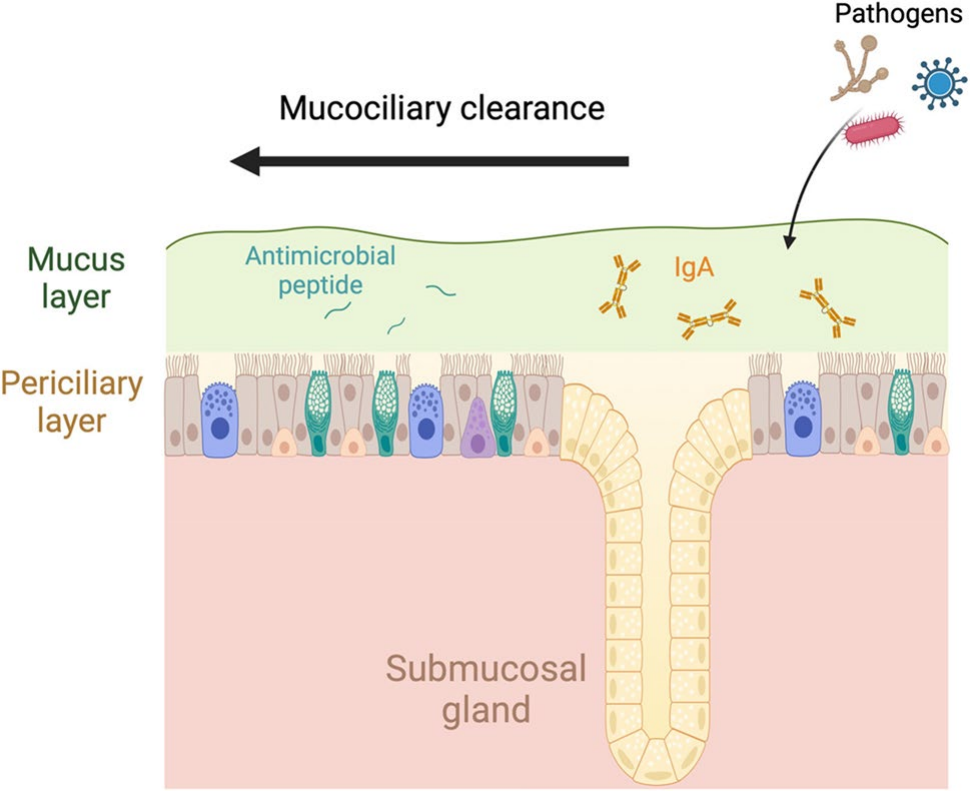
Mucus-mediated defense mechanisms. Mucus is mainly produced by secretory cells such as goblet cells and club cells. Submucosal glands also contribute substantially to the production of mucus. Its sticky nature prevents pathogens from invading the airway epithelium. Then, ciliated cells move mucus to extrude the captured foreign substances out of the body. This coordinated mechanism of mucus and epithelium is mucociliary clearance (MCC). In addition, mucus contains antimicrobial peptides and IgA and functions as a chemical barrier

In addition to the above barrier mechanism based mainly on cell adhesion, there is another defense mechanism mediated by mucus production in the respiratory system (Fig. 2). The airway epithelium, from nasal to bronchioles, is coated with mucus, which is a viscous and gel-like secretion. The characteristic viscous, elastic, and adhesive properties of mucus are mainly attributed to mucins. Although many genes encoding mucins have been reported, MUC5AC and MUC5B are the predominant mucins in the airways [25]. Through its adhesive nature, mucus captures inhaled allergens, pathogens, and harmful substances, preventing these particles from reaching deeper into the lungs. Furthermore, this mucus layer is transported in a coordinated manner towards the oral cavity to facilitate the removal of these captured particles from the body, a process known as mucociliary clearance (MCC). The continuous and synchronized beating of the ciliated cells mediates this movement.

MUC5AC is primarily found in the proximal airways, including the trachea, bronchi, and bronchioles, but not in the distal bronchioles. It is predominantly synthesized by goblet cells. MUC5B is the dominant mucin in the superficial epithelium and glands across the respiratory tract, including the distal airways. Submucosal glands (SMGs), which are beneath the cartilaginous airways of the human lung, primarily secrete MUC5B along with a lesser amount of MUC5AC. These glands are composed of serous cells, secretory cells, myoepithelial cells, and goblet cells. SMGs are essential secretory structures that contribute to airway defense, mucus production, and antimicrobial protection in the lungs. Their dysfunction can have significant implications for lung health and contribute to the pathogenesis of respiratory diseases. In healthy individuals, MUC5B is more prevalent than MUC5AC, and maintaining a proper balance between MUC5AC and MUC5B is crucial for effective MCC. Pathological increases in MUC5AC have been linked to the onset, progression, and heightened exacerbation risk of COPD [26]. Although controversial results have been reported on the increase or decrease of MUC5AC and MUC5B due to various methods of assessing mucin, there is no doubt that the pathophysiology of cystic fibrosis (CF) is related to the viscous mucus secreted from the epithelial surface of the respiratory and intestinal tract [27, 28].

Interestingly, it has been reported that the ratio of MUC5AC to MUC5B is altered in asthmatic patients with increased production of MUC5AC [29]. Furthermore, the absence of MUC5B may result in impaired mucociliary clearance by persistent inflammation [30]. These findings underscore the close relationship between the function of the mucus layer and various pulmonary diseases.

The periciliary layer (PCL), distinct from the overlaying mucus layer, is a thin, watery layer surrounding the cilia. Composed of a low-viscosity fluid containing membrane-associated mucins (MUC1, MUC4, and MUC16) and other molecules, including glycolipids, the PCL allows unimpeded ciliary movement. This feature is critical for propelling the mucus layer and trapped particles out of the respiratory system, highlighting its essential role in effective MCC [31]. The significance of ciliary movement is apparent when considering primary ciliary dyskinesia (PCD), a genetic disorder characterized by chronic respiratory tract infections and abnormal ciliary structure and function. In PCD, these cilia are either structurally abnormal or paralyzed, leading to a buildup of mucus and bacteria, resulting in frequent infections of the lungs, ears, sinuses, and throat. Treatment typically focuses on managing symptoms and preventing lung damage, often requiring regular airway clearance, antibiotics for infections, and careful monitoring [32].

### Chemical barriers

In addition to mucins, the mucus contains water, salts, lipids, immunoglobulin A (IgA), and antimicrobial proteins. These antimicrobial proteins, including defensin, lysosome, and lactoferrin, directly neutralize or destroy bacteria, viruses, and fungi by disrupting microbial cell membranes or interfering with essential microbial processes. Beyond their direct antimicrobial functions, they modulate the immune responses, ensuring a balance between effective defense and tissue protection. Moreover, IgA also provides a multifaceted defense, balancing effective protection against pathogens [33]. IgA is an antibody class present predominantly on the surfaces of mucous tissues, including the lungs. It exists in its secretory form, secretory IgA (sIgA), whose polymeric structure, often dimeric, enhances its avidity and functional capacity in mucus. Produced by tissue-resident memory B cells, plasmablasts, and plasma cells within the respiratory mucosa, IgA is transported across epithelial cells into the mucus by the polymeric immunoglobulin receptor (pIgR), predominantly expressed on secretory cells [34–36]. Dimeric IgA (dIgA) binds to pIgR on the basolateral surface of airway epithelial cells. Following binding, the IgA-pIgR complex is internalized through endocytosis and transported across the epithelial cell to the apical surface in a process known as transcytosis. During transcytosis, pIgR undergoes proteolytic cleavage, resulting in the formation of the secretory component (SC). Upon release into the mucus layer, SC remains bound to dIgA, thus forming sIgA. Once bound to pathogens, IgA can neutralize them, preventing them from entering cells. Additionally, IgA has anti-inflammatory properties, ensuring that immune responses do not inadvertently damage lung tissue [37].

Associations between sIgA and various respiratory diseases, such as COPD, asthma, and COVID-19, have been observed. Decreased expression of pIgR and degradation of sIgA may lead to loss of sIgA in COPD small airways [36]. On the other hand, patients with severe COPD have increased IgA expression in lung lymphoid follicles [38]. In asthma patients, the expression of pIgR in bronchial epithelial cells is reduced due to IL-4Rα-mediated signaling [39]. Recent findings indicate that IgA autoantibodies targeting pulmonary surfactant B and C in severe COVID-19 patients may contribute to respiratory failure, suggesting that IgA acts not only protectively but also pathologically [40]. Elucidating the precise role of IgA in the airways will be vital to understanding the pathogenesis of many respiratory diseases.

## Airway epithelial cell barriers

AECs play a critical role in establishing the physiological barrier in the lungs. Recent advances in analytical technologies have revealed that AECs are a more heterogeneous population and constitute a more complex network than previously assumed [41–45]. Here, we summarize the characteristics of subpopulations of AECs.

### Basal cells

Basal cells exhibit a columnar or cuboidal shape and are firmly anchored to the basement membrane through specialized structures known as hemidesmosomes. They can be identified based on the expression of p63 and keratin 5 (KRT5). Functionally, they are stem cell–like and can self-renew and differentiate into various epithelial cell subtypes, playing a crucial role in maintaining the integrity of the epithelial barrier and facilitating repair and regeneration after injury.

Recent studies have suggested that basal cell abnormalities may contribute to the development of several respiratory diseases. The comparison of RNA-seq data of basal cells between smokers and nonsmokers showed that COPD risk genes identified in GWAS are upregulated by smoking [46]. Other findings suggest that sensing apoptotic cells by a TAM receptor tyrosine kinase Axl on basal cells is important for tracheal basal cell expansion, cell cycle reentry, and symmetric cell division and is involved in the pathogenesis of COPD [47]. Meanwhile, single-cell RNA sequencing (scRNA-seq) analysis of lungs from IPF patients identified an aberrant basal cell population that co-expressed basal epithelial, mesenchymal, senescence, and developmental markers [48]. Another group used single-cell cloning technology to generate a library of basal stem cells from lungs derived from IPF patients [49]. Among these clones, a clone that transformed normal lung fibroblasts into pathogenic myofibroblasts in vitro was identified. Interestingly, this clone resembled the genetic profile of the abnormal basal cell population identified by scRNA-seq described above [48, 49]. The relationship between basal cells and allergic pathology has also been the focus of interest. Based on scRNA-seq data of airway epithelial cells derived from patients with chronic rhinosinusitis or asthma, two populations of basal cells with different gene expressions have been reported [50, 51]. These two populations correspond to differentiation stages, with the less mature population characterized by high expression of TP63. Furthermore, IL-4 and IL-13 have been shown to regulate basal cell stem function in vitro [50]. In addition, because of their progenitor function, basal cells have attracted attention for their application in regenerative medicine [52, 53].

### Ciliated cells

Ciliated cells are abundant in the large and medium airways and are crucial in moving mucus, which traps debris and pathogens, out of the airways. Each ciliated cell has numerous cilia that extend into the mucus. These cilia are anchored to the cytoskeleton and move in a coordinated manner. They are terminally differentiated and can originate from secretory cells or basal cells. Notch signaling pathways regulate the differentiation of ciliated cells. When Notch signaling is inhibited, it promotes the differentiation of ciliated cells [54]. Also, forkhead box protein J1 (FOXJ1) is a master regulator of ciliogenesis. Its expression is essential for the differentiation of ciliated cells and the formation of motile cilia. Of clinical importance, ciliated cells have been reported to be involved in viral infections and asthma [6, 8, 55]. Rhinovirus C (RV-C), the predominant cause of the common cold, is infectious via cadherin-related family member 3 (CDHR3) in the host [56]. The expression of CDHR3 is shown to be mainly restricted to ciliated cells [57]. Moreover, CDHR3 has been identified as a susceptibility gene for asthma, especially in young children [58]. These findings suggest a strong association between rhinovirus infections, CDHR3, and the development of asthma [55, 58].

### Club cells

Club cells, formerly known as Clara cells, are distinctive dome-shaped cubical cells found in the small airways and characterized by the expression of secretoglobin family 1A member 1 (Scgb1a1), also known as Clara cell secretory protein (CCSP). Their differentiation depends on the transforming growth factor-b receptor Alk5 (activin receptor-like kinase 5). They secrete a variety of substances, the most notable being Scgb1a1. This protein has anti-inflammatory and immunosuppressive properties by suppressing various pro-inflammatory cytokines [59]. In an ALI (air–liquid interface) culture using airway epithelial cells derived from COPD patients, the supplementation of Scgb1a1 was shown to regulate IL-8 release by cigarette smoke extract [60]. Immunostaining for Scgb1a1 in the airways was reduced in COPD patients and decreased with increasing severity of COPD, suggesting the association between club cells and the pathogenesis of COPD [61]. It has also been reported that Scgb1a1 in bronchoalveolar lavage (BAL) is decreased in asthma patients [62]. Of interest, low mRNA expression levels of Scgb1a1 in airway epithelial cell brushings in asthmatic patients have been shown to correlate with poor clinical outcomes [63]. In addition, club cells possess the ability to differentiate into both ciliated cells and goblet cells. Moreover, in cases where basal cells are injured or lost, club cells can differentiate into basal cells [64]. Thus, because of the high plasticity of lung epithelial cells, it was difficult to identify the origin cell of the tumor. However, using a lineage-tracking mouse model and scRNA-seq, it has been shown that the causative cells of lung adenocarcinoma are club cells and AT2 cells [65].

### Goblet cells

Goblet cells are characterized by their densely packed mucin granules and surfactant proteins. Their primary function is to produce and secrete mucus. In particular, MUC5AC is mainly synthesized by goblet cells. With ciliated cells, they play a crucial role in facilitating effective MCC. Goblet cells are derived from club cells through the activation of SAM pointed domain containing ETS transcription factor (SPDEF) and forkhead box A3 (FOXA3). In several lung diseases, such as asthma and COPD, there is an increase in the number of goblet cells, a condition known as goblet cell hyperplasia [66, 67]. This can cause coughing and wheezing due to excessive mucus production. In particular, allergic inflammation and goblet cells are closely related, and IL-13 induces goblet cell hyperplasia and metaplasia [68, 69].

### Pulmonary neuroendocrine cells

Pulmonary neuroendocrine cells (PNECs) can be found either as individual isolated cells or organized in small clusters known as neuroendocrine bodies (NEBs) throughout the conducting airways, especially near the respiratory tree branch. While not being neurons themselves, PNECs are innervated by both the sympathetic and parasympathetic nervous systems. PNECs can monitor airway oxygen and respiratory status and quickly release various substances, including neurotransmitters such as gamma-aminobutyric acid (GABA), calcitonin gene–related peptide (CGRP), bombesin, and serotonin [70]. These neurotransmitters produced by PNECs have been reported to induce goblet cell hyperplasia and ILC2 activation [14, 71]. Thus, PNECs are considered to be important in the coordination of the epithelial, immune, and nervous systems, facilitating respiratory homeostasis.

### Tuft cells

Tuft cells, also known as brush cells, are expressed in various tissues, including the respiratory and gastrointestinal tracts. Their distinctive morphology, characterized by a tuft of microvilli extending into the mucosal lumen, enables them to sense the extracellular environment. They can detect changes in the local chemical composition and transmit signals to nearby cells [72, 73]. While the exact function of tuft cells in the lung is still being elucidated, it has been reported that they can release IL-25 and cysteinyl leukotrienes (CysLTs), which synergistically contribute to type 2 inflammation in the lung [74].

### Ionocytes

Recently, pulmonary ionocytes were identified as a rare cell type using scRNA-seq of human bronchial epithelial cells and mouse tracheal epithelial cells [41, 45]. Ionocytes co-express forkhead box l1 (FOXL1), multiple subunits of the vacuolar-type H^+^-ATPase (V-ATPase), and cystic fibrosis transmembrane conductance regulator (CFTR). One of the most notable characteristics is their high expression of CFTR, which encodes an anion channel critical for maintaining the hydration and pH of the airway surface liquid. Thus, they may play an essential role in MCC by cooperating with ciliated cells and secretory cells. Moreover, because mutations in the CFTR gene are responsible for CF, ionocytes have attracted attention as a therapeutic target for CF [75]. However, it has been recently suggested that the function of CFTR in human airways is mainly carried out by secretory cells, as ionocytes are a small population [76]. On the other hand, the regulatory function of CFTR-mediated chloride differs between cell types, with secretory cells involved in the secretion of chloride and ionocytes in absorption [77]. Analysis using the transgenic ferret models suggests that there are at least three subtypes of ionocytes [78]. Further analysis of ionocytes is expected to clarify the pathogenesis of CF and other airway diseases.

### Alveolar cells

The alveolar epithelium consists of two types of epithelial cells: alveolar type1 (AT1) cells and alveolar type 2 (AT2) cells [16]. AT1 cells are highly specialized for gas exchange and barrier function, characterized by their flat, thin, and squamous morphology. This distinctive shape allows AT1 cells to efficiently cover about 95% of the alveolar surface. AT1 cells, together with capillary endothelium, form the alveolar-capillary barrier, otherwise known as the air-blood barrier. This incredibly thin barrier is crucial not only for efficient gas exchange between the bloodstream carbon dioxide and airborne oxygen but also for separating the bloodstream from foreign pathogens.

AT2 cells are cuboidal cells with apical microvilli and lamellar bodies. Their most important physiological role is the synthesis and secretion of pulmonary surfactant. Pulmonary surfactant reduces surface tension within the alveoli, preventing alveolar collapse during exhalation and preserving alveolar structure for efficient breathing. Besides surfactants, AT2 cells produce various cytokines, chemokines, growth factors, and antimicrobial peptides, which play roles in inflammation, immune responses, and tissue repair.

In addition, AT2 cells serve as progenitor cells for alveolar epithelium. When the alveolar epithelium is injured, AT2 cells can undergo self-renewal and differentiate into AT1 cells, contributing to tissue repair and regeneration [79]. Wnt signaling may be involved in maintaining the stem cell properties of AT2 cells [80–82]. Furthermore, their regenerative potential is likely to inform the development of novel therapies for COPD [83] and pulmonary fibrosis [84, 85].

Two recent reports of scRNA-seq analyses of human distal airways have identified AT0 cells and respiratory airway secretory (RAS) cells. AT0 cells, originated from AT2 cells, can differentiate into AT1 cells or terminal and respiratory bronchiole secretory cells [43]. In contrast, RAS cells can unidirectional differentiate into AT2 cells through Notch and Wnt signaling [86]. These cells, which are capable of differentiation, may exhibit phenotypic alterations with age or in response to pathological conditions; thereby, their function and pathology need to be analyzed under a broader range of conditions.

## Immunological barriers by pattern recognition receptors

The first line of defense in the lungs is not only the physicochemical barriers but also the innate immune system. The innate immune system provides immediate, non-specific responses to threats. Pattern recognition receptors (PRRs) on innate immune cells detect conserved structures on pathogens, namely pathogen-associated molecular patterns (PAMPs). PRRs also recognize damage-associated molecular patterns (DAMPs) from stressed or damaged host cells. Upon recognition, these receptors initiate various immune responses, including inflammation, phagocytosis, and the production of cytokines and chemokines, thereby inducing the adaptive immune system. Common types of PRRs include Toll-like receptors (TLRs), NOD-like receptors (NLRs), retinoic acid-inducible gene-I (RIG-I)–like receptors (RLRs), and C-type lectin receptors (CLRs). Notably, PRRs are expressed in AECs as well as innate immune cells [87].

### TLRs

TLRs, which are membrane-bound receptors, can be found on the cell surface or in intracellular vesicles like endosomes. In humans, there are ten distinct TLRs (TLR1 to TLR10), each recognizing specific PAMPs. TLR1, TLR2, TLR4, TLR5, TLR6, and TLR10 are localized to the plasm membrane, where they can recognize and respond to extracellular pathogens. For instance, TLR4 recognizes lipopolysaccharide (LPS) from gram-negative bacteria, and TLR5 detects bacterial flagellin. On the other hand, TLR3, TLR7, TLR8, and TLR9 are localized in endosomes, where they can detect intracellular nucleic acids. TLR3 senses double-stranded RNA, often associated with viral infections. TLRs are expressed in a wide range of cell types, enabling a coordinated and broad response to potential threats [88–90].

### NLRs

NLRs are a group of cytoplasmic PRRs containing three domains: a C-terminal leucine-rich repeat (LRR) domain, a nucleotide-binding domain (NBD), and an N-terminal effector domain. The LRR domain is involved in ligand recognition. NBD is highly conserved among NLRs and is crucial in forming NLR complexes or inflammasomes. The N-terminal effector domain varies among different NLR family members and determines the downstream signaling pathway and interactions with other proteins. There are different types of effector domains, including caspase and activation and recruitment domain (CARD), pyrin domain (PYD), baculoviral inhibitor of apoptosis repeat (BIR) domain, and transactivator domain (AD). When a ligand binds to the LRR domain, it induces a conformational change in the NLR. This change leads to the exposure or activation of the NBD, initiating the formation of protein complexes called inflammasomes. The formation of inflammasomes results in the activation of caspase-1 and the cleavage of inactive precursor forms of IL-1β and IL-18 into their active forms [91].

### RLRs

RLRs, which are a class of cytoplasmic PRRs, consist of three members: RIG-I, melanoma differentiation-associated gene 5 (MDA5), and laboratory of genetics and physiology 2 (LGP-2) [92]. RLRs contain DExD/H box RNA helicase domains that are central to their RNA sensing function. RIG-I recognizes short double-stranded RNA (dsRNA) with 5′-triphosphates, often found in RNA viruses, while MDA5 detects longer dsRNA. When RIG-I or MDA5 binds to RNA ligands, they undergo conformational changes that expose their N-terminal CARD, initiating downstream signaling through interactions with adaptor proteins like mitochondrial antiviral signaling (MAVS). This signaling cascade leads to the production of antiviral cytokines and the activation of immune responses. On the contrary, LGP-2 lacks the CARD and cannot directly initiate antiviral signaling but acts as a regulatory protein that modulates the signaling of RIG-I and MDA5 in a context-dependent manner.

### CLRs

CLRs are numerous and include both membrane-bound and soluble receptors. They recognize carbohydrate structures, primarily on pathogens, through their carbohydrate recognition domains (CRDs). Membrane-bound CLRs are mainly found on myeloid cells, including dendritic cells and macrophages. Soluble CLRs, like mannose-binding lectin (MBL), circulate in the extracellular environment and can activate the complement system. Upon ligand binding, CLRs lead to immune responses like phagocytosis and cytokine production [89, 93]. Some CLRs, such as Dectin-1, sense specific pathogens like fungi [94].

## Interactions of epithelial and immune cells in the lung barrier

In recent years, deeper analysis of cellular networks, such as crosstalk between epithelial and immune cells, has become feasible. A contributing factor to this advancement is the progress in transcriptome technologies at the single-cell level, exemplified by scRNA-seq. Furthermore, initially, single-cell transcriptomes had difficulties in data integration and interpretation due to problems such as batch effect, but these problems are being overcome due to large datasets, powerful computational resources, and advances in learning algorithms.

Single-cell transcriptomics offers significant advantages, including the identification of rare cell populations like ionocytes and the estimation of cell differentiation processes through trajectory analysis [41, 45]. Furthermore, these techniques have enabled the inference of intercellular networks by utilizing information on ligand-receptor interactions [43, 86]. These advancements are being applied to a variety of respiratory diseases.

Specifically, scRNA-seq conducted on nasopharyngeal and bronchial samples collected from patients with moderate to severe COVID-19 has been reported [95]. In these patients, secretory cells exhibited significantly higher expression of chemokines such as CXCL1, CXCL3, CXCL6, and CXCL17 compared to controls, suggesting an enhancement in the mobilization of neutrophils, T cells, and mast cells. Furthermore, in severe cases compared to moderate, there was a stronger interaction between epithelial and immune cells, with immune cells, including inflammatory macrophages, being more activated. Thus, this interaction may contribute to the exacerbation of infection. Importantly, infected epithelial cells showed upregulation of the SARS-CoV-2 entry receptor ACE2, which was associated with interferon signaling in immune cells. These findings suggest that intercellular links are important not only in the severity of infection but also in the establishment of infection.

Utilizing a human model of localized asthma exacerbation by bronchoscopic segmental allergen challenge, a comprehensive analysis of the lower airway mucosa of allergic asthmatics and allergic non-asthmatics using scRNA-seq has been conducted [96]. In response to allergens, asthmatic airway epithelium was highly dynamically upregulating genes involved in matrix degradation, mucus metaplasia, and glycolysis while failing to induce the injury-repair and antioxidant pathways observed in controls. The study revealed a Th2 cell-mononuclear phagocyte-basal cell interactome unique to asthmatics, driven by interactions between TNF family members and type 2 cytokines from Th2 cells. This pathogenic cellular network in asthmatics may override protective injury-repair responses and drive asthma pathobiology.

In addition to the single-cell transcriptome, the advancement of three-dimensional (3D) culture systems, represented by organoids, experimental models that closely recapitulate human physiology, allow further analysis in a coordinated manner [43, 86, 97]. Fetal lung organoids derived from fetal lung bud tips provide significant insights into lung development. In humans, SOX2 and SOX9 have been identified as progenitor markers, and their maintenance requires EGF, FGF, and WNT signaling, as well as the inhibition of BMP and TGF-β [98]. The interaction between epithelium and immune cells was also shown to play an important role in lung development using fetal lung organoids [99]. In immunohistochemistry of fetal lungs, immune cells were found surrounding lung progenitor cells. Therefore, the expression of cytokine receptors in progenitor cells was evaluated, and candidates for interacting cytokines between epithelial and immune cells were identified. Among various cytokines, the supplementation of IL-1β in fetal lung organoids resulted in decreased expression of SOX9 and increased expression of TP63. These findings suggest that myeloid cells, widespread throughout the lungs, produce IL-1β during early lung development, which induces epithelial stem cell differentiation into mature basal cells.

Besides organoids, precision-cut lung slices (PCLS) have emerged in respiratory research as a 3D culture method [100]. PCLS are live tissue preparations that encompass all resident cell types, including smooth muscle cells, epithelial cells, and fibroblasts. These cells maintain intercellular interactions and cell–matrix relationships within the complex structure of the lung, making them suitable for analysis of cellular networks through single-cell transcriptomics.

Recently, the utilization of human PCLS (hPCLS), single-cell transcriptome analysis, and deep learning-based query-to-reference mapping has been reported as a powerful research platform for elucidating the pathogenesis of lung fibrosis and facilitating drug development [101]. In this study, fibrosis was induced in hPCLS from nonfibrotic human lung tissue by adding a pro-fibrotic cytokine mix, and the scRNA-seq data obtained from the fibrosis-induced hPCLS were merged with single-cell data from a cohort of pulmonary fibrosis patients. Furthermore, single-cell architectural surgery (scArches), which is a deep learning strategy for mapping single-cell datasets to a reference atlas, was employed to map the obtained data to the Human Lung Cell Atlas, thereby validating an ex vivo model of fibrosis. Additionally, analyses of cell morphology and intercellular networks were conducted through micro-CT staging of hPCLS and patient tissues. The pathways of fibrogenesis and healing processes were evaluated by administering antifibrotic drugs to fibrosis-induced hPCLS. As demonstrated by this cutting-edge research, the analysis of cellular networks in lung physiology and pathogenesis is expected to accelerate with the advancements in transcriptome data analysis, human-like experimental systems, and artificial intelligence technology.

## Immunological barriers by myeloid cells

The lungs contain numerous immune cells that maintain respiratory homeostasis. However, the immune system is not solely composed of immune cells but also interacts with non-immune cells such as AECs and neural cells. This section summarizes the subtypes of innate immune cells in the lung, along with their characteristics and contribution to the defense mechanisms.

### Alveolar macrophages

Alveolar macrophages (AMs) are the most abundant immune cells in the airway lumen and are crucial components of the respiratory innate immune systems [102]. They express the high levels of CD11c, Siglec-F, and CD169 and lack CD11b [103]. GM-CSF is required for the differentiation and maturation of AMs. Recently, it has been reported that AT2 cell–derived GM-CSF plays a nonredundant and critical role in establishing the postnatal AM population and maintaining AMs in adult lungs, emphasizing a critical link between epithelial and immune cells [104]. AMs have the phagocytic function to clear inhaled pathogens, dead cells, and foreign airborne particles, maintaining the health of the alveolar environment. In addition, they can also produce pro-inflammatory cytokines via PRRs and induce adaptive immune responses. Concerning these pro-inflammatory properties, paralysis of immune function due to poor phagocytosis of AMs has been reported following severe infections. Increased SIRP1a expression on AMs during the early stages of paralysis directly impairs phagocytosis, and SIRP1a acts as a tyrosine kinase receptor, triggering the induction of an immunosuppressive microenvironment [105].

On the other hand, they can produce anti-inflammatory molecules, such as IL-10 and TGF-β, to prevent excessive inflammation and tissue damage. A subset of AMs, forming connexin 43 (CX43)–containing gap junction channels with the alveolar epithelium, communicates immunosuppressive signals to reduce lipopolysaccharide-induced lung inflammation [106]. Besides immune regulation, they contribute to the homeostasis of pulmonary surfactants [102]. Pulmonary alveolar proteinosis (PAP) is a disease characterized by the abnormal accumulation of surfactants in the alveoli, leading to respiratory failure. Autoimmune PAP, which constitutes the majority of PAP, is initiated by the presence of anti-GM-CSF autoantibodies in patients’ sera [107]. These antibodies result in the functional impairment of AMs, which is the underlying etiology of PAP [108]. Notably, it has been reported that the administration of GM-CSF can effectively ameliorate respiratory conditions in patients with PAP [109].

### Interstitial macrophages

Interstitial macrophages (IMs) are a distinct population of macrophages located in the lung interstitium or parenchyma. They express high levels of CD11b but lack Siglec-F, which distinguishes them from AMs [103]. While they also have phagocytic functions, IMs are more involved in modulating immune responses and tissue repair. In addition, IMs have been shown to produce IL-10 spontaneously [110].

### Eosinophils

Eosinophils are involved in immune responses to parasitic, bacterial, and viral infections, as well as in the maintenance of homeostasis. In response to stimuli, they release granule proteins, including cytotoxic major basic protein (MBP), eosinophil cationic protein (ECP), eosinophil peroxidase (EPX), Galectin-10, lipid mediators, and many cytokines. These granule proteins help eliminate parasites, bacteria, and viruses, while excessive release may cause damage to surrounding tissue and cells. For example, MBP promotes histamine release from mast cells and triggers a cascade of type 2 inflammation. Furthermore, eosinophils could secrete type 2 cytokines, such as IL-4, IL-9, and IL-13. Thus, eosinophils are hallmark cells in allergic asthma and other allergic lung diseases, contributing to bronchoconstriction, mucus production, and inflammation [111, 112]. Eosinophil-targeted treatments are crucial for managing eosinophilic asthma and similar conditions. Corticosteroids are commonly used, either inhaled or systemic, to counter eosinophilic inflammation. In addition, biologics have been developed to reduce eosinophils [113]: mepolizumab and reslizumab target IL-5, suppressing eosinophil development and activity, whereas benralizumab specifically binds to the IL-5 receptor to kill eosinophils by antibody-dependent cell-mediated cytotoxicity. Dupilumab inhibits IL-4 and IL-13, both essential for eosinophils to accumulate in inflamed tissues.

Moreover, recent findings have indicated that eosinophils are not a homogenous population [114]. In the murine lung, resident eosinophils (rEos), characterized by Siglec-F^int^CD62L^+^CD101^lo^, are present at a steady state. However, an additional subset of eosinophils as inflammatory eosinophils (iEos), characterized by Siglec-F^hi^CD62L^−^CD101^hi^, emerges alongside rEos during allergic inflammation caused by allergen inhalation. Importantly, iEos and rEos possess different functional profiles: rEos can inhibit allergen-loaded dendritic cells’ maturation and pro-Th2 function, whereas iEos promotes allergic inflammation [115]. Identifying and understanding specific eosinophil subtypes can lead to the development of more targeted therapies, minimizing side effects and improving treatment efficacy.

### Neutrophils

Neutrophils constitute a pivotal component of the innate immune system, eliminating pathogens, primarily bacteria and fungi. Neutrophils recognize invading pathogens and execute their antimicrobial defense functions through phagocytosis and the release of diverse cytokines and chemokines, thereby orchestrating the recruitment of inflammatory immune cells [116]. Moreover, neutrophils form neutrophil extracellular traps (NETs), which are composed of DNA, histones, and antimicrobial proteins. NETs serve as a physical barrier to trap and kill pathogens. After the initial response, neutrophils undergo apoptosis and are also removed by macrophages to limit excessive inflammation. Thus, while neutrophils play an indispensable role in immune defense, they can be implicated in the pathogenesis of various diseases. For instance, type 2–low asthma is associated with pronounced neutrophil infiltration [117]. Furthermore, their dysregulated responses can contribute to the development of COPD and acute respiratory distress syndrome (ARDS) [118]. Therefore, the appropriate regulation of neutrophil activity is essential for maintaining lung homeostasis.

### Dendritic cells

Dendritic cells (DCs) play a crucial role in the immune system by capturing inhaled pathogens and allergens and subsequently migrating to lymph nodes, where they present intracellularly processed antigens to antigen-specific T cells. These DCs encompass diverse subsets that exhibit specialized functions based on their anatomical localization and their roles in pathogen recognition, thereby contributing to various immune responses. While the categorization of DC subsets in the lung remains a subject of debate, they can generally be classified into two major categories: plasmacytoid DCs (pDCs) and conventional DCs (cDCs), with the latter further subdivided into cDC1 and cDC2 [119]. pDCs primarily serve as producers of type 1 interferons, primarily upon TLR7 and TLR9 stimulation, which are vital in combating viral infections. However, the pathogenicity of pDCs deserves attention, as type 1 interferons have also been implicated in the pathogenesis of autoimmune diseases such as systemic lupus erythematosus and psoriasis [120, 121].

Distinct markers differentiate cDC1 from cDC2, with the former expressing CD103 and the latter expressing CD11b. During viral infections, cDC1s assume a critical role in the induction of effector CD8^+^ T cells within the lung by facilitating cross-presentation via MHC class I molecules. Conversely, cDC2s are pivotal in generating central memory CD8^+^ T cells. In the context of asthma pathogenesis, cDC1s contribute to the suppression of Th2 and Th17 immune responses through IL-12 production, while cDC2s promote Th2 and Th17 immune responses in response to challenges with house dust mites (HDM) via receptor engagement, such as Dectin-1 [122]. In summary, dendritic cells in the lung encompass various subsets that perform specialized functions, impacting immune responses to pathogens/allergens and their involvement in disease pathogenesis, thereby highlighting their significance in pulmonary immunology [123, 124].

## Immunological barriers by innate lymphoid cells

Innate lymphoid cells (ILCs) are a group of innate immune cells that are primarily involved in defending the body against infections, particularly at mucosal surfaces like the lungs, gut, and skin. ILCs lack specific antigen-specific receptors like T cell receptors (TCRs) or B cell receptors (BCRs), leading to their ability to respond quickly to stimuli, particularly cytokines. ILCs can be classified into three distinct functional groups, namely ILC1s, ILC2s, and ILC3, similar to the classification of CD4^+^ T cells, with each corresponding to Th1, Th2, and Th17 cells, respectively [125].

### ILC1s

ILC1s are characterized by the expression of T-box transcription factor 21 and the production of IFN-γ, a key cytokine involved in the defense against intracellular pathogens. They share some functional similarities with Th1 cells, which also produce IFN-γ. In mice infected with the H1N1 influenza virus, ILC1s are activated and respond quickly to release IFN-γ and TNF-α [126]. Additionally, during the Sendai virus infection, ILC1s were identified as the primary source of IFN-γ production in the early phase [127]. Although the roles of ILC1s in the lung are not fully understood, the increased percentage of ILC1s in the blood of COPD patients suggests that ILC1s are involved in the pathogenesis of COPD [128].

### ILC2s

**Fig. 3:**
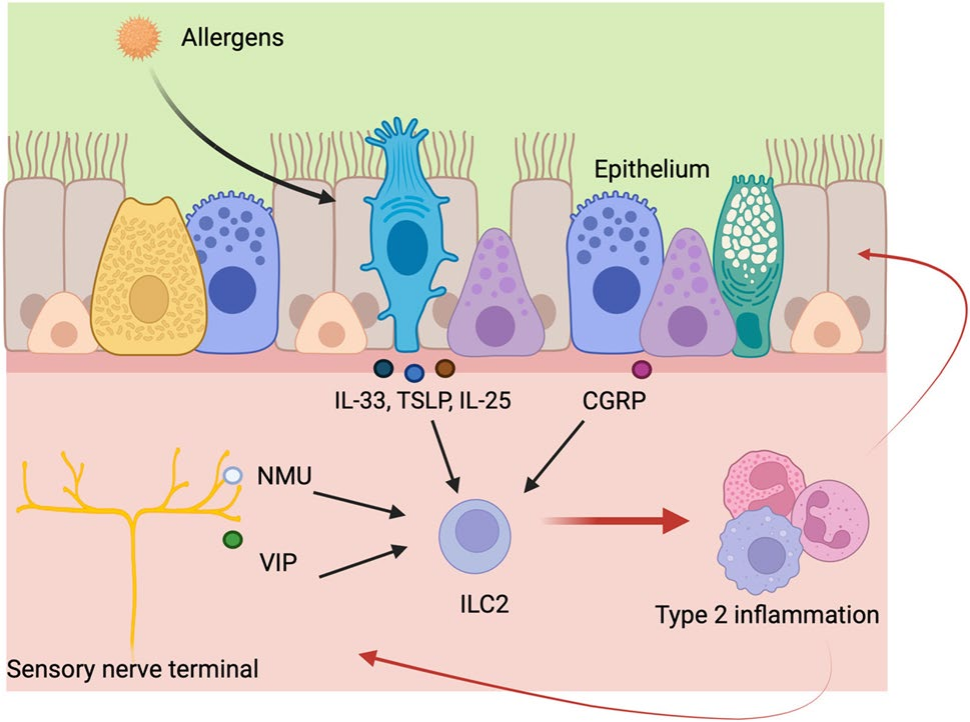
The induction of type 2 inflammation through the interactions between epithelial cells, neurons, and immune cells. When allergens invade the respiratory epithelium, tuft cells release cytokines such as IL-25, and pulmonary neuroendocrine cells (PNECs) produce calcitonin gene–related peptide (CGRP), which can activate ILC2s. Epithelial cells release IL-33 and TSLP upon stimulation and/or damage, activating ILC2s. In addition, neuromedin U (NMU) and vasoactive intestinal peptide (VIP) from sensory neurons stimulate ILC2s to release cytokines, leading to type 2 inflammation. The induction of type 2 inflammation mobilizes various immune cells, which also act on the nervous system and epithelium to enhance inflammation

The master regulator of ILC2s is a transcription factor called GATA-binding protein-3 (GATA3), similar to Th2 cells. Neuropilin-1 (NRP1), which is induced postnatally and sustained by lung-derived transforming growth factor beta-1 (TGF-β1), has been reported to be a tissue-specific marker of lung ILC2s. ILC2s promote pulmonary inflammation by secreting type 2 cytokines, including IL-5, IL-13, and IL-4, in response to epithelial-derived alarming cytokines such as IL-33, TSLP, and IL-25 (Fig. 3) [129]. Many reports indicate that ILC2s have a pathogenic role in allergic inflammation [130]. In a mouse model of asthma, Th2 cells and ILC2s are the main sources of IL-5 and IL-13 production in the lung. In addition to allergic inflammation, ILC2s are also involved in tissue repair through amphiregulin production. Recently, the relationship between ILC2s and neural cells is an emerging area of research that has revealed intriguing connections between the immune and nervous systems [10]. Neuropeptides, which are signaling molecules produced by neurons, can influence the activity of ILC2s. For instance, vasoactive intestinal peptide (VIP) can directly activate ILC2s, leading to their production of IL-5. Then, IL-5 released from ILC2s stimulates neurons to produce more VIP, establishing a positive feedback loop [11]. Moreover, neuromedin U (NMU), produced by mucosal neurons, has been shown to activate ILC2s [12]. Upon binding to its receptor (neuromedin U receptor 1) on ILC2s, NMU could stimulate ILC2s to produce cytokines. This relationship between NMU and ILC2s has been reported not only in allergic airway inflammation but also in respiratory syncytial virus (RSV) infection. In response to RSV, pulmonary neurons secrete NMU in a TLR4- and TLR7-dependent manner, activating ILC2s via NMU and thus exacerbating airway inflammation [13]. In addition, it has been reported that calcitonin gene–related peptide (CGRP) produced by pulmonary neuroendocrine cells (PNECs) promotes cytokine production from ILC2s [14].

### ILC3s

ILC3s are characterized by the expression of retinoic acid receptor-related orphan receptor γt (RORγt) and release IL-17, IL-22, and GM-CSF in response to IL-23 and IL-1b. They are the predominant ILC subpopulation in human lungs, whereas ILC2s are the most abundant in murine lungs. Through the production of IL-22 and IL-17, ILC3s are involved in defense against various pathogens. In addition, they are the main producers of IL-22 in the lung and are essential for maintaining epithelial homeostasis and tissue repair. On the other hand, it has been reported that ILC3s act pathogenically in obesity-related asthma, suggesting diverse roles for ILC3s in the lungs [131, 132].

## Concluding remarks

The importance of epithelium as a barrier mechanism in the lungs has been well established. Their central role was thought to be a physical mechanism through cell adhesion and a protective mechanism through mucus production. Indeed, it has been demonstrated that dysfunction of these barrier mechanisms is involved in many pathological conditions, suggesting their importance. Recently, the role of AECs as initiators of immune responses has been recognized. Furthermore, the close relationship between epithelium, neurons, and immune cells is becoming apparent. Further studies are needed to determine the physiological role of this crosstalk and its involvement in the pathogenesis of various lung diseases.

## Data Availability

No original data was generated for this review article.
